# Altered cognition and anxiety in adolescent offspring whose mothers underwent different-pattern maternal sleep deprivation, and cognition link to hippocampal expressions of *Bdnf* and *Syt-1*

**DOI:** 10.3389/fnbeh.2022.1066725

**Published:** 2022-12-08

**Authors:** Ru-Meng Wei, Yue-Ming Zhang, Yun Li, Qi-Tao Wu, Ya-Tao Wang, Xue-Yan Li, Xue-Wei Li, Gui-Hai Chen

**Affiliations:** ^1^Department of Neurology (Sleep Disorders), The Affiliated Chaohu Hospital of Anhui Medical University, Hefei, Anhui, China; ^2^Department of Neurology, The First Affiliated Hospital, Hengyang Medical School, University of South China, Hengyang, Hunan, China

**Keywords:** sleep deprivation, spatial learning and memory, anxiety, BDNF, synaptotagmin-1

## Abstract

**Background:**

Inadequate sleep during pregnancy negatively affects the neural development of offspring. Previous studies have focused on the continuous sleep deprivation (CSD) paradigm, but the sleep pattern during late pregnancy is usually fragmented.

**Objective:**

To compare the effects of CSD and fragmented sleep deprivation (FSD) in late pregnancy on emotion, cognition, and expression of synaptic plasticity-related proteins in offspring mice.

**Methods:**

Pregnant CD-1 mice were either subjected to 3/6 h of CSD/FSD during gestation days 15–21, while those in the control group were left untreated. After delivery, the offspring were divided into five groups, i.e., control (CON), short or long CSD (CSD3h, CSD6h), and short or long FSD (FSD3h, FSD6h). When the offspring were 2 months old, the anxiety-like behavior level was tested using the open field (OF) and elevated plus maze (EPM) test, and spatial learning and memory were evaluated using the Morris water maze (MWM) test. The expression of hippocampal of *brain-derived neurotrophic factor* (*Bdnf*) and *synaptotagmin-1* (*Syt-1*) was determined using RT-PCR and western blotting.

**Results:**

The CSD6h, FSD3h, and FSD6h had longer latency, fewer center times in the OF test, less open arms time and fewer numbers of entries in the open arms of the EPM, longer learning distance swam and lower memory percentage of distance swam in the target quadrant in the MWM test, and decreased BDNF and increased Syt-1 mRNA and protein levels in the hippocampus. Compared to the CSD6h, the FSD3h and FSD6h had longer distance swam, a lower percentage of distance swam in the target quadrant, decreased BDNF, and increased Syt-1 mRNA and protein levels in the hippocampus.

**Conclusion:**

The results suggested that maternal sleep deprivation during late pregnancy impairs emotion and cognition in offspring, and FSD worsened the cognitive performance to a higher extent than CSD. The observed cognitive impairment could be associated with the expression of altered hippocampal of *Bdnf* and *Syt-1* genes.

## Introduction

Inadequate sleep during pregnancy is a common phenomenon, and epidemiological studies have demonstrated that approximately half of the population of pregnant women is dissatisfied with the quality of sleep (Sedov et al., 2018). Owing to the influence of estrogen and progesterone levels, psychological state, and fetal development, pregnant women are susceptible to sleep disorders, including insomnia, sleep-disordered breathing, and restless leg syndrome, resulting in inadequate sleep (Mindell et al., 2015). Irregular contractions, increased fetal movements, leg cramps, frequent urination, and dyspnea in late pregnancy lead to frequent awakenings, decreased slow-wave sleep, and decreased rapid eye movement (REM) sleep (Wilson et al., 2011; Izci-Balserak et al., 2018). Therefore, sleep patterns in pregnant women usually exhibit fragmentation.

To date, various sleep deprivation (SD) paradigms have been applied in basic research, including the gentle stimulation method (Longordo et al., 2009), the treadmill method (Ward et al., 2009), and the modified multiple platform method (Machado et al., 2004). These SD paradigms are divided into total SD and selective SD. For instance, the modified multiple-platform method selectively deprives REM sleep, based on the principle that muscle tension declines when the sleep stage changes from non-REM to REM sleep (Kim et al., 2021). In contrast, the gentle stimulation method is a continuous SD (CSD) paradigm that completely deprives all sleep stages by physically touching or knocking on the cage (Vecsey et al., 2013). However, none of these SD paradigms can properly simulate the sleep-deprived patterns in late pregnancy, as they merely deprive specific sleep stages, while the most frequent sleep disturbance during pregnancy is sleep fragmentation (Khalyfa et al., 2014). It is, therefore, necessary to use an appropriate SD paradigm for exploring the effects of maternal SD (MSD) on the behaviors of offspring and underlying mechanisms.

There have been some reports of fragmented SD (FSD) paradigms, which randomly interrupt sleep by arousing the animals at various specific time intervals, resulting in fractured sleep. The FSD paradigm designed for rats includes a 2-min cycle of 30 s-on and 90 s-off for 24 or 72 h on a treadmill, ensuring that the animals awaken during the 30 s-on intervals (Tartar et al., 2006). A relatively mild protocol is designed for mice, which includes 2-min repetitive cycles of 10 s-on and 110 s-off during the light-on phase that lasts for 2 months, by using an electronically controlled running rotor (Xie et al., 2020). After undergoing these procedures, the rodents exhibit impaired spatial learning and memory and increased anxiety-like behaviors. It seems that these paradigms are applicable to simulate the characteristics of fragmented sleep in clinical settings.

Insufficient sleep during pregnancy is thought to play an adverse role in the growth and development of the offspring (Chang et al., 2010; Zhao et al., 2014). Experimental studies on rodents have demonstrated that offspring whose mothers experienced SD during pregnancy exhibit impaired cognition, altered anxiety-like behaviors, decreased male sexual behaviors, and dysfunctional metabolism (Alvarenga et al., 2013; Radhakrishnan et al., 2015; Peng et al., 2016; Trzepizur et al., 2017). As paradigm heterogeneity can result in different emotional and cognitive outcomes, it is necessary to compare the effects of SD during late pregnancy on the emotional and cognitive behaviors of offspring using different procedures (CSD/FSD).

The hippocampus is a key region of the brain that is involved in processing cognitive and emotional information (Anacker and Hen, 2017), whose functions can be impaired by adverse stresses in early life (Grigoryan and Segal, 2016). Indeed, SD during pregnancy has adverse effects on the hippocampus of the offspring, including reduced neurogenesis and altered synaptic plasticity, which results in emotional and cognitive deficits (Zhao et al., 2014; Yu et al., 2018). The impaired synaptic plasticity could be related to the altered levels of synaptic plasticity-associated proteins in the hippocampus. However, the alterations in the synaptic proteins of mice whose mothers suffered from different SD during pregnancy are poorly studied.

Brain-derived neurotrophic factor (BDNF) is an important regulator of synaptic plasticity in the central nervous system and plays a key role in the formation of learning and memory (Autry and Monteggia, 2012). After binding to tropomyosin-related kinase B (TrkB) receptors, BDNF affects the downstream signaling pathways to regulate long-term potentiation (LTP) and synaptic transmission, which affects synaptic plasticity (Leal et al., 2014). Previous studies have demonstrated that exposure to stress during early life, including maternal separation and prenatal inflammation, can decrease the hippocampal levels of BDNF in adulthood (Schaafsma et al., 2017; Menezes et al., 2020). Synaptotagmin-1 (Syt-1), an important presynaptic vesicle protein, is the main fast-phase Ca^2+^ receptor during the exocytosis of synaptic vesicles and plays an important role in synaptic plasticity (Chapman, 2002). Previous studies have demonstrated that adult CD-1 mice exposure to inflammation during pregnancy exacerbates age-related learning and memory decline accompanied by increased levels of Syt-1 (Li et al., 2016). These findings suggest that the two synaptic plasticity-associated proteins, BDNF and Syt-1, play an important role in learning and memory. However, it remains to be understood whether the effects of SD during pregnancy on emotion and cognitive function in offspring are mediated *via* BDNF and Syt-1.

Therefore, the present study aimed to explore whether different SD paradigms during late pregnancy affect the emotion and hippocampus-dependent spatial learning and memory in adolescent CD-1 mice offspring, and if so, whether the effects are mediated *via* alterations in the expression levels of Syt-1 and BDNF in the hippocampus of offspring mice.

## Methods

### Animals

CD-1 mice (7–8 weeks) were purchased from Beijing Vital River Laboratory Animal Company (SPF grade). Adaptive feeding was performed for 1 week before experimentation. Mating was performed in the cage at 21:00 h at a male:female ratio of 1:2. The vaginal plug was checked for the following days at 7:00 h, and the day the vaginal plug was observed was designated as day 0 of gestation (gd 0). Pregnant females were housed individually in cages in a feeding environment under a 12-h light/dark cycle (lights on at 8:00 h and off at 20:00 h), at a temperature of 22–25°C, relative humidity of 50 ± 5%, and had *ad libitum* access to food and water. The pregnant mice were randomly divided into five groups during late gestation (gd 15–21), with 10 pregnancy mice in each group, and subjected to short or long CSD (3/6 h) or FSD (3/6 h), while the control group did not receive any intervention. The delivery day was designated as postnatal day 0 (PND0). The offspring of these pregnant mice were used as the study subjects and were noted accordingly: long CSD (CSD6h), long FSD (FSD6h), short CSD (CSD3h), short FSD (FSD3h), and control (CON) groups. The offspring were reared up to adolescence (2-month-old) after birth (Spear, 2000; Kota et al., 2011; Zhang Z. Z. et al., 2022). Finally, a total of 16 mice, including 8 males and 8 females, were included in each group for behavioral analyses, western blotting, and RT-PCR experiments. The experimental procedures were approved by the Laboratory Animal Committee of Anhui Medical University.

### Sleep deprivation

**Figure 1:**
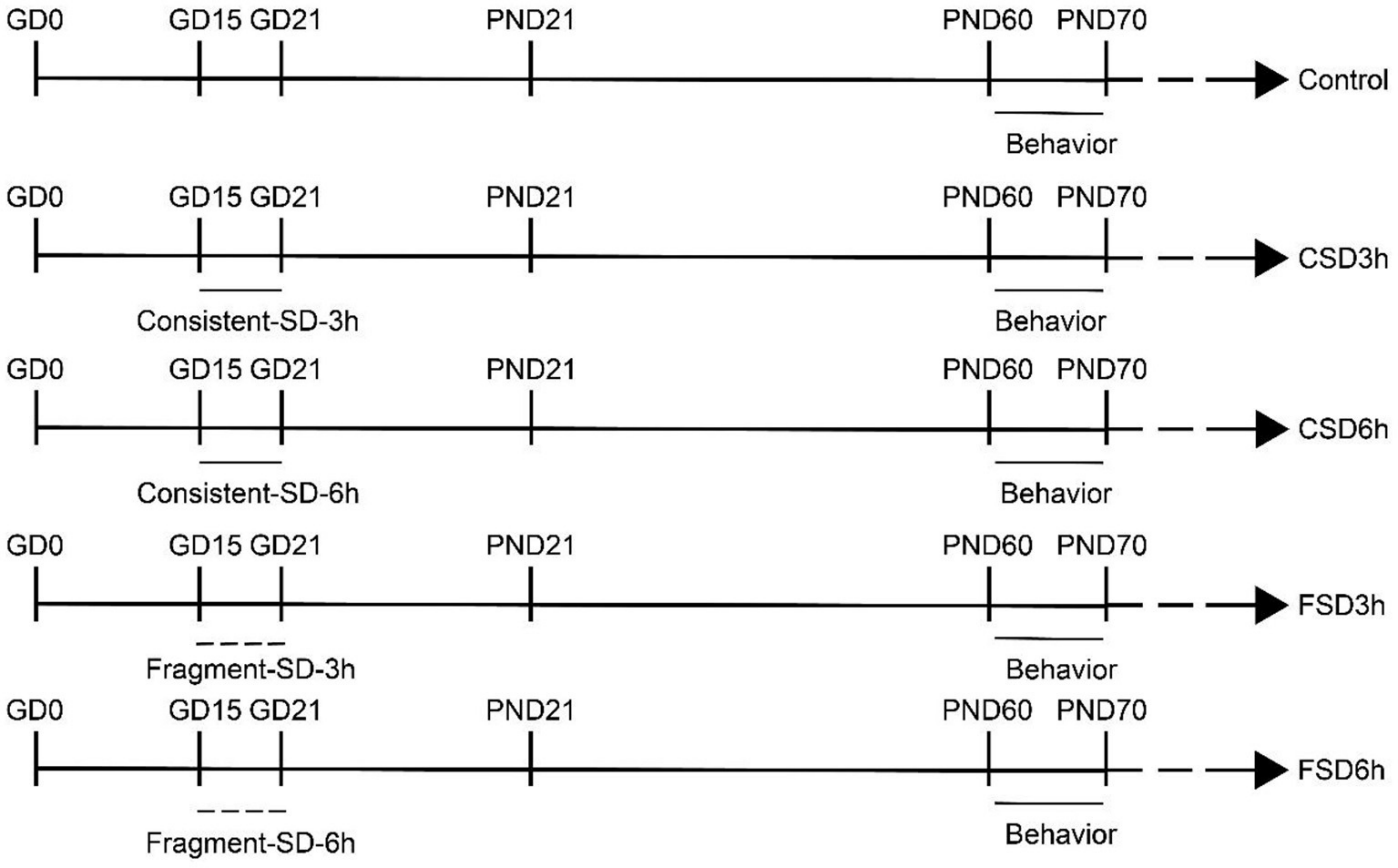
Timeline of the experiment (refer to experimental protocols for details).

Sleep deprivation was performed using a special apparatus (BW-NSD404, Shanghai Bio-will Co, Ltd) for 3 h (12:00 h−15:00 h) or 6 h (12:00 h−18:00 h) per day. In the FSD, the repetitive cycle of the SD apparatus was set to 10 s-on and 110 s-off periods with 30 interruptions/h (Xie et al., 2020), while the device moved uninterruptedly at 0.5 m/min in the CSD (Zhang Y. M. et al., 2022). Food and water were made available throughout the period of SD (Figure 1).

### Vaginal smear

The vaginal smear method was used to assess the estrous cycle of female mice, which is divided into four phases: proestrus, estrus, metaestrus, and diestrus (Ng et al., 2010). The female mice were selected to start behavioral experiments in the diestrus due to the lesser effect of estrogen during this period (Wessels et al., 2015). The vagina of mice was gently rinsed 3–4 times with 20 μl of stroke physiological saline solution in a 200-μl pipette, the vaginal rinsing solution was evenly applied to the slides, and the slides were dried naturally and fixed with anhydrous ethanol at room temperature for 10 min. The slides were gently rinsed with running water, gently shaken off, and stained with hematoxylin stain for 5 min; the floating color was washed off in the water. The unbound hematoxylin was eluted with 1% hydrochloric acid ethanol treatment for 5 s and then the slides were gently rinsed in water. The slides were re-stained with ammonia for 30 s to return to blue, gently rinsed in water, and then shaken off. The slides were stained with 0.5% eosin for 30 s; the slides were gently rinsed in water and then air-dried, sealed, and photographed with a confocal microscope (Olympus FV3000, Japan) to observe the cell composition of the vaginal smears (Supplementary Figure 3).

### Open field test

The open field (OF) test (Tong et al., 2015) was used for evaluating locomotor activity and anxiety-like behaviors and was performed at PND61. The experimental device consisted of a wooden box without a lid and a black inner wall (internal dimensions of 81 × 81 × 28 cm^3^). The bottom of the box was equally divided into 16 squares of dimensions 20 × 20 cm. A colored toy was placed in the center to stimulate the curiosity and exploratory activity of the mice. The mice were individually placed in a corner square facing the wall and were permitted to freely explore the environment for 5 min. After each test, the urine and feces of each mouse were removed, and 75% alcohol was used for avoiding interference in the subsequent experiments. The ANY-Maze software (Stoeling, USA) was used for automatic calculations and analyses of latency (time spent to leave the first square) and center time (time spent in the four center squares).

### Elevated plus maze test

The elevated plus maze (EPM) test (Chen et al., 2022) was used for studying the anxiety-like behaviors of the offspring and was performed at PND62. The equipment appeared like a cross and consisted of two open arms and two closed arms (30 cm in length and 5 cm in width each) with a center square (5 × 5 cm^2^). The equipment was elevated to a height of 80 cm above the floor. The mice were individually placed in the center square area, facing one of the open arms. The maze was cleaned with 75% ethanol after the test was complete for each mouse. The ANY-Maze software (Stoeling, USA) was used to analyze the time spent in the open arms and the number of entries into the open arms within 6 min.

### Morris water maze test

The spatial learning and memory abilities of the 2-month-old mice offspring were assessed by the Morris water maze (MWM) test over a period of 7 days, with 16 mice per group. The tests were carried out from PND63 to P69. The protocol used in this study has been previously reported (Wu et al., 2020). The water maze consisted of an underwater platform (10 cm in diameter and 24 cm in height) and a circular black tank (150 cm in diameter and 30 cm in height) filled with water (20–22°C, 25 cm in depth). The periphery of the tank was surrounded by a white curtain from the ceiling to the floor, in which three black cardboard shapes (square, triangle, and circle) served as spatial cues. The formal experiment was divided into two parts: the learning phase (the navigation task) and the memory phase (the probe trial task). In the learning phase, the mice were randomly placed in the water from the four quadrants with their heads facing the wall of the pool for each test and were allowed to rest on the platform for 30 s regardless of whether they climbed onto the platform within the specified time (60 s) or were guided by the experimenter to the platform. A total of four tests were conducted each day with a 15 min interval, and the experiment lasted for 7 days. In the memory phase, the experiment was performed on the last day of the learning period, after 2 h of the last trial. Briefly, the platform was removed, and the mice were placed in the water from the opposite quadrant of the target quadrant and allowed to explore freely for 60 s. Swimming distance, speed of finding the platform in the learning phase, and the percentage of distance swam in the target quadrant in the memory phase were analyzed using the ANY-Maze software (Stoeling, USA).

### Tissue preparation

The CD-1 mice were euthanized after giving the proper anesthesia (2% Sodium pentobarbital) at PND71. The brain tissues were rapidly removed from the skull and cut along with the midsagittal plane on ice. The cerebral cortex was carefully dissected to expose the hippocampus and separate the hippocampus from the cerebral cortex and the surrounding brain tissue. Finally, the hippocampal tissue was carefully collected and snap-frozen in liquid nitrogen followed by transfer to −80°C for various biochemical analyses.

### Western blotting

The hippocampus was stripped and grounded thoroughly, following which 600 μl of RIPA cell lysis buffer containing 0.6 mM PMSF (Beyotime, P0013B) was added for lysis, and centrifuged at 12,000 rpm for 15 min. The supernatant was collected, and 5X SDS-PAGE protein loading buffer (Solarbio, S8010) was added to the collected protein samples at a ratio of 1:4. The samples were then heated in a boiling water bath for 15 min to fully denature the proteins. After the samples had cooled down to room temperature, 5–10 μl of the protein sample was directly loaded into each of the loading wells of the gel used for SDS-PAGE. Electrophoresis was performed at a constant pressure, at 80 volts, for ~1 h. The gels were then placed over pre-cut filter papers and a PVDF membrane (Millipore, IPVH00010) of the same size as the adhesive strip that had been previously soaked in methanol for 2–3 min and immersed in a transfer buffer for 5 min. Constant flow transfer membranes with a transfer time of 20 and 45 min were used for BDNF and Syt-1, respectively. The protein membrane was then immediately placed in a pre-prepared western wash solution and rinsed for 5 min for washing off the transfer solution from the membrane. The western blotting solution containing 5% skimmed milk powder was added, and the membrane was slowly shaken on a shaker and blocked for 2 h at room temperature. The membrane was subsequently incubated with the rabbit anti-BDNF (1:1000; Abcam, Cambridge, UK) and rabbit anti-Syt-1 (1:1000; Bioss, Beijing, China) primary antibodies, and the horseradish peroxidase (HRP) labeled goat anti-rabbit IgG (Zsbio, ZB-2301) secondary antibody, according to the manufacturer's instructions. The protein bands were analyzed using Image J software (Media Cybernetics, USA) for calculating the relative expression of BDNF and Syt-1.

### Real-time fluorescence-based quantitative PCR

The RNA was reverse transcribed into cDNA using a reverse transcription kit (TaKaRa, RR047A), and the cDNA was used as a template for fluorescence quantification. The PCR mixture comprised 5 μl of 2 × SYBR Green Mixture, 1 μl of forward primer, 1 μl of reverse primer, 1 μl of cDNA, and 2 μl of RNase-Free water. The reaction conditions included a single cycle of pre-denaturation at 95°C for 1 min, denaturation at 95°C for 20 s, and extension at 60°C for 1 min for a total of 40 cycles. The relative content of the mRNA was calculated using the 2^−Δ*ΔCt*^ method. The sequences of the primers used for PCR are enlisted in Table 1.

**Table 1:** Primer sequences used in PCR.

**Gene**	**Amplicon size (bp)**	**Forward primer (5^′^ → 3^′^)**	**Reverse primer (5^′^ → 3^′^)**
*β-actin*	120	AGTGTGACGTTGACATCCGT	TGCTAGGAGCCAGAGCAGTA
*BDNF*	94	TTACTCTCCTGGGTTCCTGA	ACGTCCACTTCTGTTTCCTT
*Syt-1*	149	GTCCTTCTAGTCGTGACCTG	GCCTGATCCTTCATGGTCTT

### Statistical analyses

Statistical analysis was performed using GraphPad Prism version 8.0. The data followed a normal distribution and was expressed as mean ± standard error of the mean (SEM), and normality was examined with the Shapiro–Wilk test. The main effect of treatment was analyzed using two-way ANOVA with treatment and sex as independent variables in all groups. The swimming distance and velocity in the MWM test were analyzed by repeated measures analysis of variance (rm-ANOVA). Two-way ANOVA was used for analyzing other data, including the percentage of distance swam in the target quadrant of the MWM, time spent in the center and latency in the OF, time spent in the open arms and the number of entries into the open arms in the EPM, and the mRNA and protein expression of BDNF and Syt-1. The correlation between all the data was determined using Pearson's correlation test. Tukey's test *post-hoc* was performed to compare the results among the groups. *P* < 0.05 was considered to be statistically significant.

## Results

### Anxiety-based tasks

#### The open-field test

**Figure 2:**
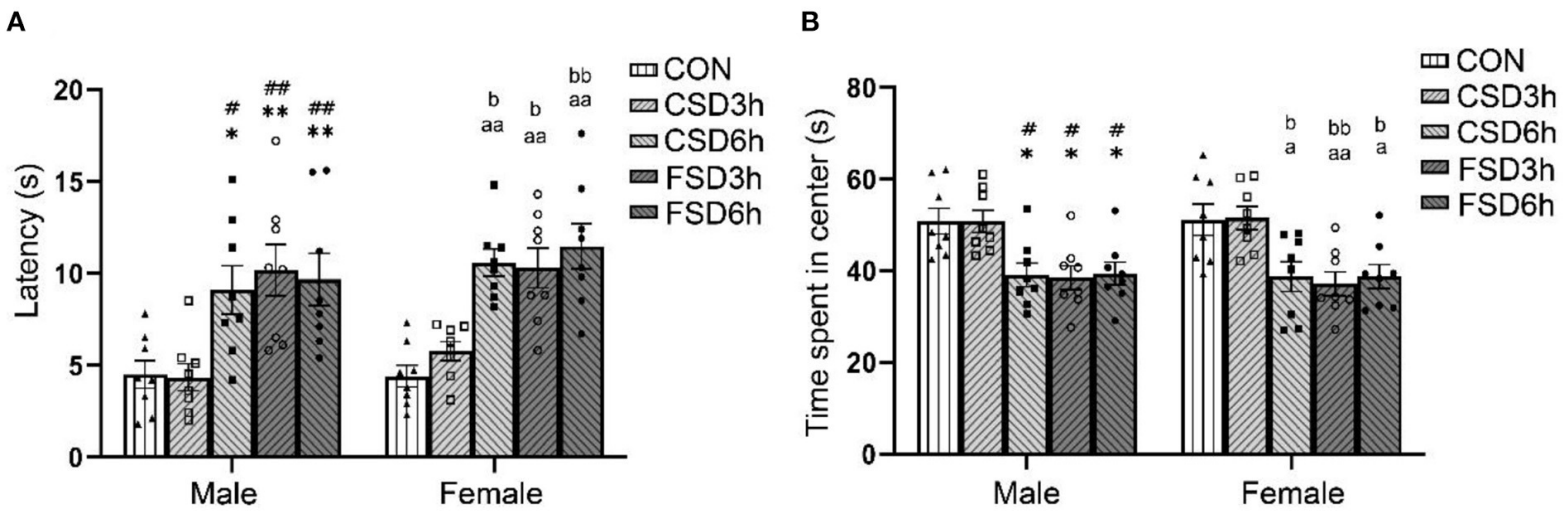
Results of the open field tests (16 mice were tested per treatment group, including 8 males and 8 females). The latency and time spent in the center in the open field are depicted graphically. A longer latency period and a shorter center time were regarded as increased anxiety-like behaviors. **(A)** Latency in the open field. **(B)** Time spent in the center. *P < 0.05 and **P < 0.01 vs. Male CON group; ^#^*P* < 0.05 and ^##^*P* < 0.01 vs. Male CSD3h group; ^a^*P* < 0.05 and ^aa^*P* < 0.01 vs. Female CON group; ^b^*P* < 0.05 and ^bb^*P* < 0.01 vs. Female CSD3h group.

The results revealed a treatment effect in the latency [*F*_(4, 70)_ = 17.102, *P* < 0.01] and center time [*F*_(4, 70)_ = 12.477, *P* < 0.01] among the five groups while there is no sex effect [latency: *F*_(1, 70)_ = 2.136, *P* = 0.148; center time: *F*_(1, 70)_ = 0.023, *P* = 0.879] and no interaction between sex × treatment [latency: *F*_(4, 70)_ = 0.349, *P* = 0.844; center time: *F*_(4, 70)_ = 0.043, *P* = 0.996]. The Tukey's test *post-hoc* analysis revealed that the mice in the CSD6h, FSD3h, and FSD6h groups exhibited longer latency (*Ps* < 0.05) and shorter center time (*Ps* < 0.05) than the mice in the CON group; however, the latency (*Ps* > 0.05) and center time (*Ps* > 0.05) were similar among these three groups. Notably, there were no significant differences in the latency and center time between the CSD3h and CON groups (*Ps* > 0.05, Figures 2A,B).

### The elevated plus maze test

**Figure 3:**
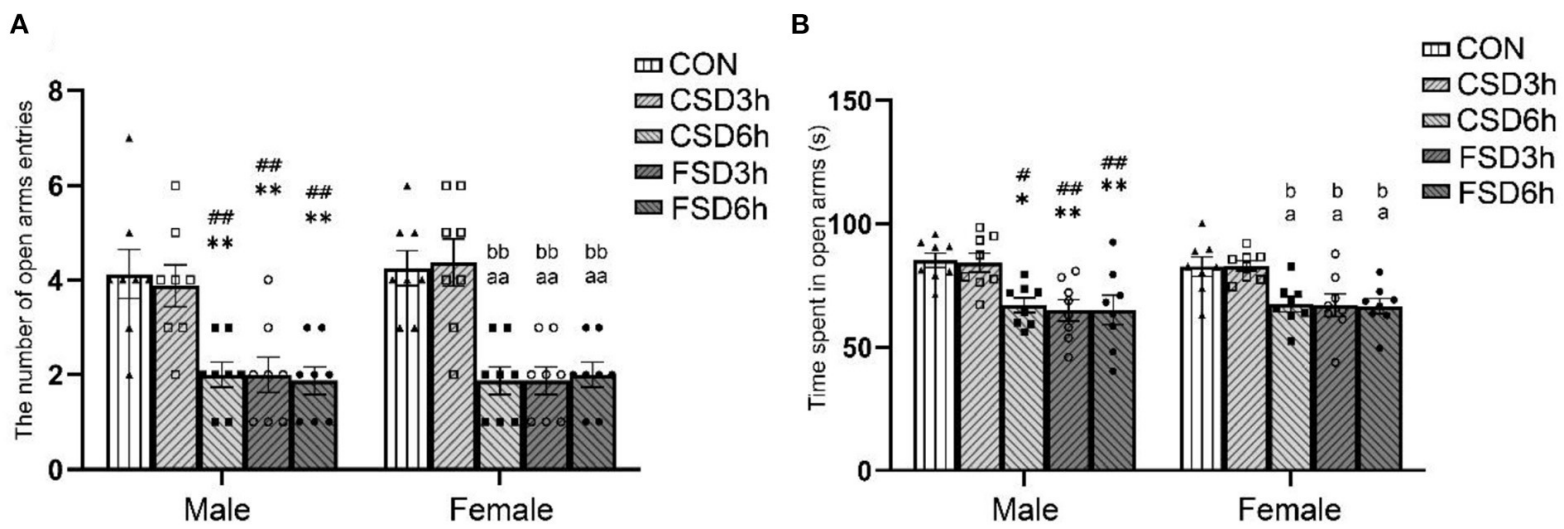
Results of the elevated plus maze test (16 mice were tested per treatment group, including 8 males and 8 females). The time spent in the center and the number of open arms entries of the elevated plus maze are depicted graphically. A shorter duration in the open arms and fewer entries into the open arms were regarded as increased anxiety-like behaviors. **(A)** Number of entries into the open arms. **(B)** Time spent in the open arms. *P < 0.05 and **P < 0.01 vs. Male CON group; ^#^*P* < 0.05 and ^##^*P* < 0.01 vs. Male CSD3h group; ^a^*P* < 0.05 and ^aa^*P* < 0.01 vs. Female CON group; ^b^*P* < 0.05 and ^bb^*P* < 0.01 vs. Female CSD3h group.

The results revealed that the SD treatments had a significant effect on time spent in the open arms [*F*_(4, 70)_ = 12.473, *P* < 0.01] and the number of open arms entries [*F*_(4, 70)_ = 21.281, *P* < 0.01], while the sex had no effect on those (time spent in open arms: *P* = 0.995; the number of open arms entries: *P* = 0.673). The interaction of sex × treatment had no significant effect on those parameters (time spent in open arms: *P* = 0.973, the number of open arms entries: *P* = 0.917). The Tukey's test *post-hoc* analysis revealed that the mice in the CSD6h, FSD3h, and FSD6h groups exhibited a shorter duration in the open arms (*Ps* < 0.05) and fewer number of entries in the open arms (*Ps* < 0.01) than the mice in the CON group, and there was a similarity in the three measures among the three groups (*Ps* > 0.05). There were no significant differences in time spent in the open arms and the number of entries into the open arms between the CSD3h and CON groups (*Ps* > 0.05, Figures 3A,B).

### Morris water maze test

#### Learning phase

**Figure 4:**
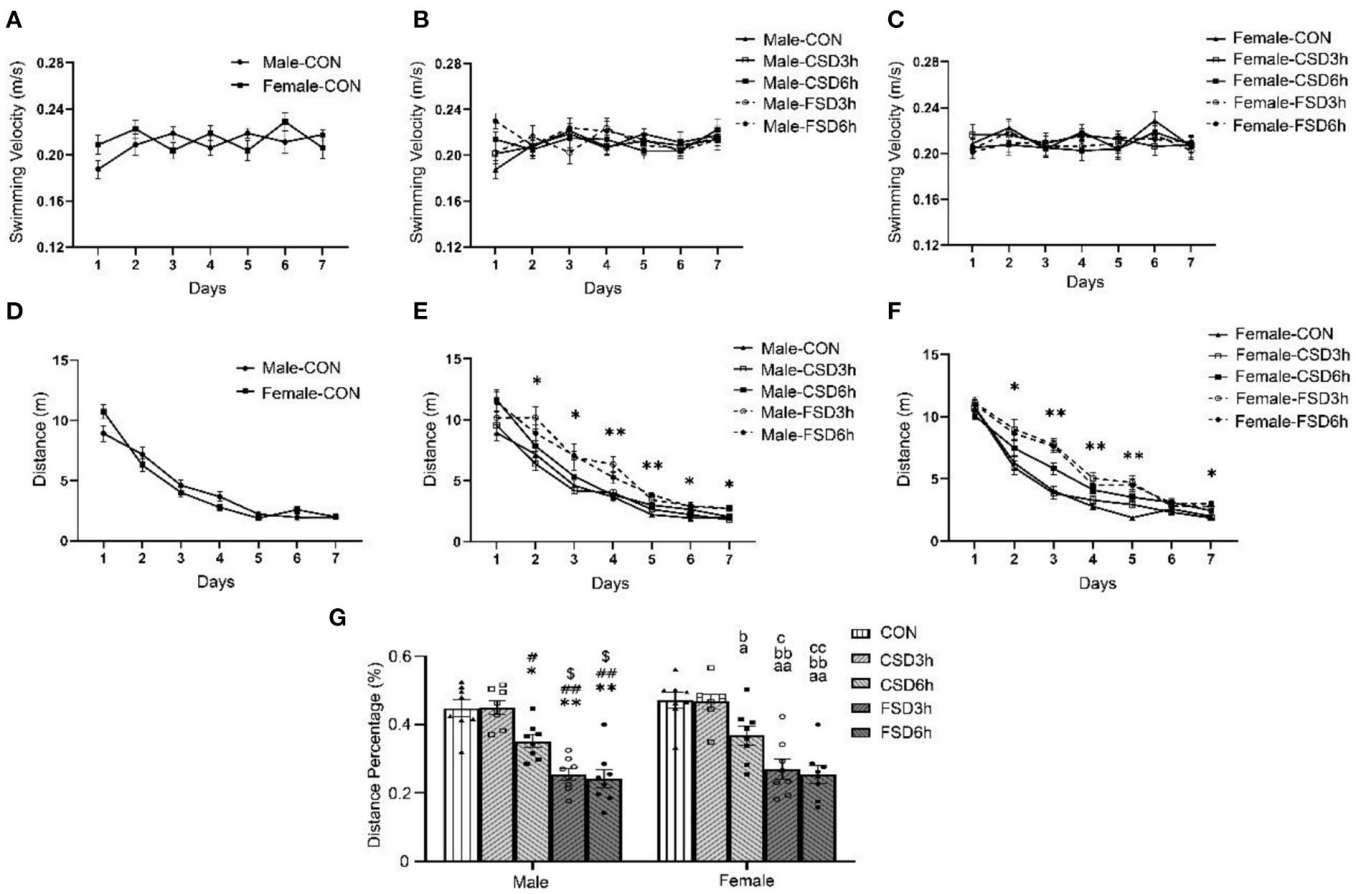
Results of the Morris water maze test (16 mice were tested per treatment group, including 8 males and 8 females). **(A–G)** The velocity and distance swam covered during the 7 days of training, and the distance percentage in the target quadrant for space exploration are depicted graphically. Longer distance swam was regarded as a reduction in learning ability, while lower percentages of distance swam were regarded as a reduction in memory ability. **(A)** CON speed, **(B)** Male speed, **(C)** Female speed, **(D)** CON distance swam, **(E)** Male distance swam, **(F)** Female distance swam, and **(G)** distance percentage. *P < 0.05 and **P < 0.01 vs. Male CON group; ^#^*P* < 0.05 and ^##^*P* < 0.01 vs. Male CSD3h group; ^$^*P* < 0.05 vs. Male CSD6h group; ^a^*P* < 0.05 and ^aa^*P* < 0.01 vs. Female CON group; ^b^*P* < 0.05 and ^bb^*P* < 0.01 vs. Female CSD3h group; ^c^*P* < 0.05 and ^cc^*P* < 0.01 vs. Female CSD6h group.

The results revealed no sex differences in swimming velocity and distance swam for the CON [velocity: *F*_(1, 14)_ = 0.535, *P* = 0.477; distance swam: *F*_(1, 14)_ = 0.017, *P* = 0.898; Figures 4A,D] and other treatment groups (CSD3h, CSD6h, FSD3h, FSD6h) (*Ps* > 0.05; Supplementary Figures 1, 2A–D). The swimming velocity was not affected by the number of days trained [Male: *F*_(6, 210)_ = 1.634, *P* = 0.139; Female: *F*_(6, 210)_ = 1.494, *P* = 0.182] and the SD treatment [Male: *F*_(4, 35)_ = 0.746, *P* = 0.568; Female: *F*_(4, 35)_ = 0.484, *P* = 0.747] (Figures 4B,C). The distance swam gradually decreased in all groups as training progressed [Male: *F*_(6, 210)_ = 199.551, *P* < 0.01; Female: *F*_(6, 210)_ = 278.352, *P* < 0.01], and the SD treatments had significant effects on the distance swam [Male: *F*_(4, 35)_ = 20.368, *P* < 0.01; Female: *F*_(4, 35)_ = 20.542, *P* < 0.01] (Figures 4E,F). A significant interaction of time × treatment on the distance swam was observed [Male: *F*_(24, 210)_ = 1.771, *P* = 0.018; Female: *F*_(24, 210)_ = 2.882, *P* < 0.01]. Tukey's test *post-hoc* analysis revealed that the mice in the CSD6h, FSD3h, and FSD6h groups swam longer distances (*Ps* < 0.05) to find a hidden platform than those in the CON and CSD3h groups. Furthermore, the FSD3h and FSD6h groups had longer distances swam (*Ps* < 0.05) than the CSD6h group, while there were no differences between the FSD3h and FSD6h groups, and between the CON and CSD3h groups (*Ps* > 0.05), in terms of distance swam.

##### Memory phase

The SD treatments [*F*_(4, 70)_ = 37.139, *P* < 0.01] had a significant effect on the percentages of distance swam in the target quadrant, while sex [*F*_(1, 70)_ = 1.301, *P* =0.258] and the interaction of sex × treatment [*F*_(4, 70)_ = 0.012, *P* = 0.999] had no effect on it. Furthermore, the percentage of distance swam in the target quadrant in the CSD6h, FSD3h, and FSD6h groups was lower than that of the CON and CSD3h groups (*Ps* < 0.05). Also, the FSD3h and FSD6h groups showed a lower percentage of distance swam in the target quadrant than the CSD6h group (*Ps* < 0.05), while there were no differences between the FSD3h and FSD6h groups, and between the CON and CSD3h groups (*Ps* > 0.05), in terms of percentage of distance swam in the target quadrant (Figure 4G).

### Effects of SD on the hippocampal expression of *Bdnf* and *Syt-1*

**Figure 5:**
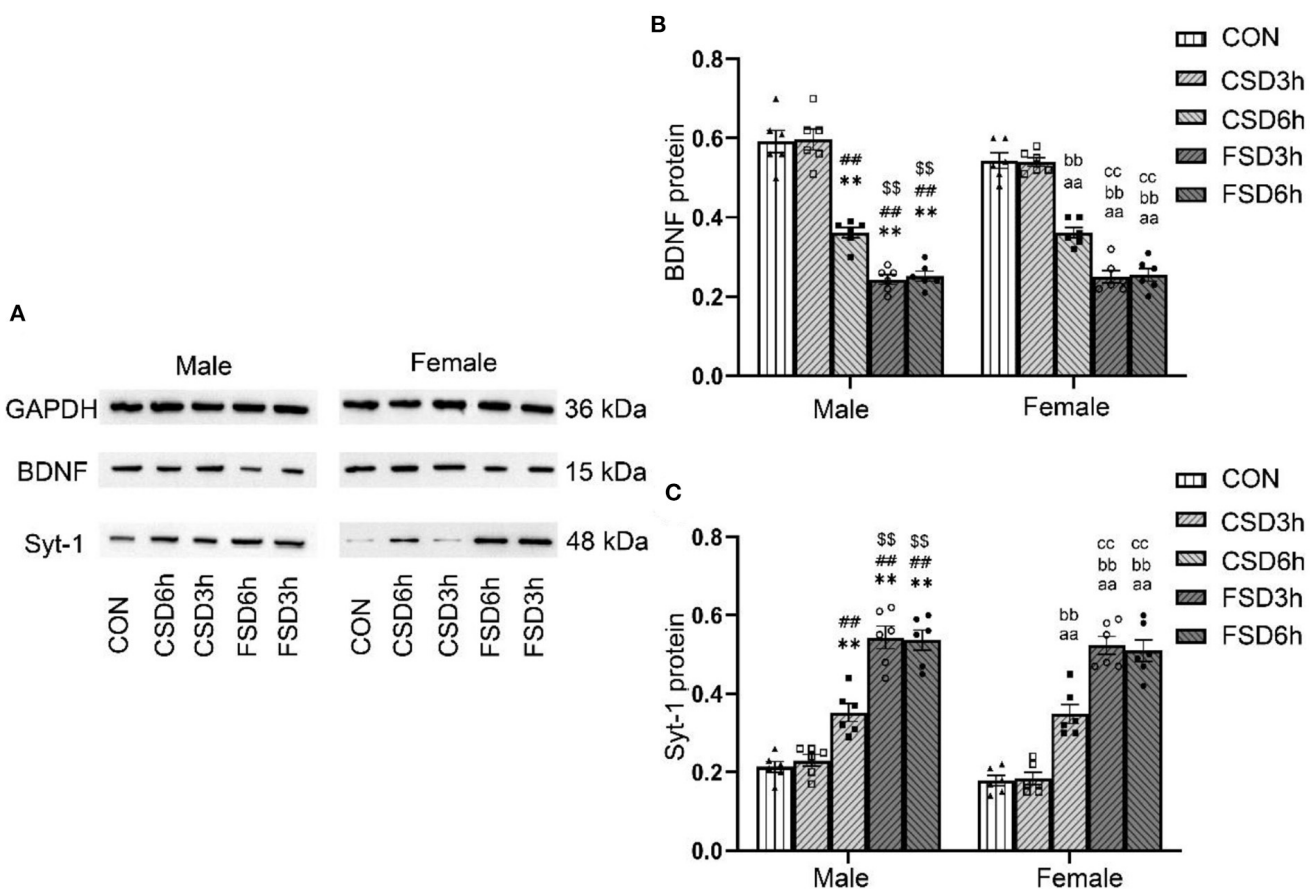
Effects of MSD on the expression of hippocampal of BDNF and Syt-1 proteins in the offspring. **(A)** The expression levels of BDNF (*n* = 12, 6 females and 6 males in each treatment group) and Syt-1 (*n* = 12, 6 females and 6 males in each treatment group) proteins in the hippocampus at 2 months. The results of protein quantification are depicted in **(B,C)**. **P < 0.01 vs. Male CON group; ^##^*P* < 0.01 vs. Male CSD3h group; ^$$^*P* < 0.01 vs. Male CSD6h group; ^aa^*P* < 0.01 vs. Female CON group; ^bb^*P* < 0.01 vs. Female CSD3h group; ^cc^*P* < 0.01 vs. Female CSD6h group.

The SD treatments had a significant effect on the hippocampal protein levels of BDNF and Syt-1 [BDNF: *F*_(4, 50)_ = 161.963, *P* < 0.01; Syt-1: *F*_(4, 50)_ = 113.377, *P* < 0.01], while the sex had no effect on those [BDNF: *F*_(1, 50)_ = 2.842, *P* = 0.098; Syt-1: *F*_(1, 50)_ = 3.665, *P* = 0.061]. No significant interaction of treatment × sex was observed on the levels of BDNF and Syt-1 protein [BDNF: *F*_(4, 50)_ = 1.495, *P* = 0.218; Syt-1: *F*_(4, 50)_ = 0.280, *P* = 0.890]. Compared to those of the CON and CSD3h groups, the CSD6h, FSD3h, and FSD6h groups had lower protein levels of BDNF (*Ps* < 0.01) and higher protein levels of Syt-1 (*Ps* < 0.01). The FSD3h and FSD6h groups also had lower protein levels of BDNF and higher protein levels of Syt-1 compared to those of the CSD6h group (*Ps* < 0.05). There were no significant differences in the protein levels of BDNF and Syt-1 between the FSD3h and FSD6h groups and between the CON and CSD3h groups (*Ps* > 0.05; Figures 5A–C).

**Figure 6:**
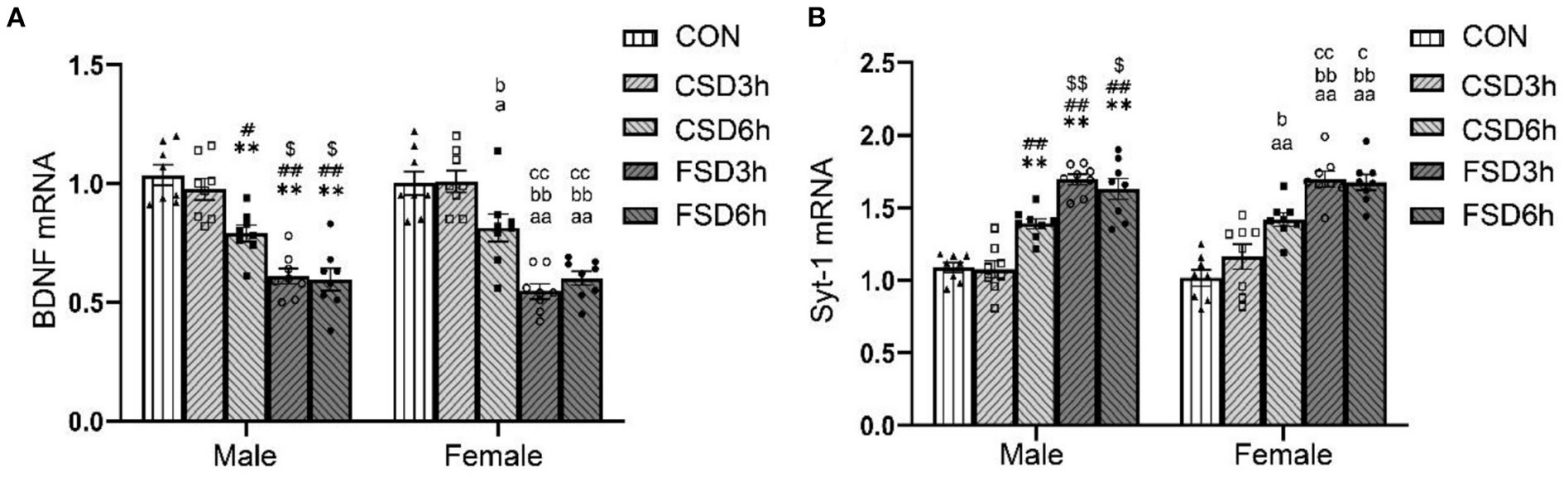
The effects of MSD on the expression of hippocampal of BDNF and Syt-1 mRNA in the offspring. The mRNA expression levels of BDNF (*n* = 16, 8 females and 8 males in each treatment group) and Syt1 (*n* = 16, 8 females and 8 males in each treatment group) in the hippocampus at 2 months. The results of mRNA quantification are depicted in **(A,B)**. **P < 0.01 vs. Male CON group; ^#^*P* < 0.05 and ^##^*P* < 0.01 vs. Male CSD3h group; ^$^*P* < 0.05 and ^$$^*P* < 0.01 vs. Male CSD6h group; ^a^*P* < 0.05 and ^aa^*P* < 0.01 vs. Female CON group; ^b^*P* < 0.05 and ^bb^*P* < 0.01 vs. Female CSD3h group; ^c^*P* < 0.05 and ^cc^*P* < 0.01 vs. Female CSD6h group.

The SD treatments had a significant effect on the hippocampal mRNA levels of BDNF and Syt-1 [BDNF: *F*_(4, 70)_ = 48.143, *P* < 0.01; Syt-1: *F*_(4, 70)_ = 57.205, *P* < 0.01], while the sex had no effect on those Syt-1 [BDNF: *F*_(1, 70)_ = 0.078, *P* = 0.782; Syt-1: *F*_(1, 70)_ = 0.287, *P* = 0.594]. No significant interaction of treatment × sex was observed on the levels of BDNF and Syt-1 mRNA [BDNF: *F*_(4, 70)_ = 0.459, *P* = 0.766; Syt-1: *F*_(4, 70)_ = 0.569, *P* = 0.686]. Similar to the expression of the protein, the CSD6h, FSD3h, and FSD6h groups had significantly lower levels of BDNF mRNA (*Ps* < 0.05) and higher levels of Syt-1 mRNA (*Ps* < 0.05) compared to those of the CON and CSD3h groups. The FSD3h and FSD6h groups also had significantly lower levels of BDNF mRNA and higher levels of Syt-1 mRNA (*Ps* < 0.05) compared to those of the CSD6h group. There were no significant differences in the levels of BDNF and Syt-1 mRNA between the FSD3h and FSD6h groups, and between the CON and CSD3h groups (*Ps* > 0.05; Figures 6A,B).

### Correlations between anxious/cognitive performance and the expression levels of BDNF and Syt-1

#### Correlation with protein levels of BDNF and Syt-1

Linear correlation analyses revealed that the hippocampal protein levels of BDNF were negatively correlated with the distance swam in the learning phase in all groups and positively correlated with the percentage of distance swam in the target quadrant in the memory phase in the CON, CSD6h, FSD3h, and FSD6h in the MWM test (*Ps* < 0.05). Additionally, the hippocampal protein levels of Syt-1 in all groups were positively correlated with the distance swam and negatively correlated with the percentage of distance swam (*Ps* < 0.05; Table 2). None of the anxiety-related indicators correlated with the hippocampal protein levels of BDNF and Syt-1 in the OF and EPM test (*P* < 0.05; Table 2).

**Table 2:** Correlation between the performance in the anxiety/cognition-related tasks and levels of hippocampal synaptic proteins [*r* (*p*)].

**Tasks**	**Indexes**	**Groups**	**Synaptic proteins**	
			**BDNF**	**Syt-1**
Open field test	Center time	CON	0.293 (0.355)	−0.002 (0.994)
		CSD3h	0.114 (0.724)	−0.031 (0.924)
		CSD6h	0.266 (0.404)	−0.477 (0.117)
		FSD3h	0.000 (1.000)	0.170 (0.598)
		FSD6h	0.166 (0.605)	0.102 (0.753)
	Latency	CON	0.292 (0.357)	−0.009 (0.977)
		CSD3h	−0.372 (0.233)	0.104 (0.748)
		CSD6h	−0.412 (0.184)	0.433 (0.160)
		FSD3h	−0.200 (0.533)	−0.004 (0.990)
		FSD6h	0.541 (0.069)	−0.258 (0.417)
Elevated plus maze test	Number of entries into the open arms	CON	0.362 (0.248)	−0.268 (0.400)
		CSD3h	0.280 (0.378)	−0.453 (0.139)
		CSD6h	0.263 (0.410)	−0.208 (0.516)
		FSD3h	−0.137 (0.672)	−0.052 (0.872)
		FSD6h	0.488 (0.107)	−0.245 (0.442)
	Time spent in the open arms	CON	0.202 (0.530)	−0.219 (0.495)
		CSD3h	0.268 (0.400)	−0.086 (0.791)
		CSD6h	−0.025 (0.937)	0.136 (0.673)
		FSD3h	0.203 (0.527)	0.230 (0.472)
		FSD6h	−0.107 (0.741)	−0.230 (0.472)
Morris water maze test	Distance swam	CON	−0.802 (0.002)^**^	0.688 (0.013)^*^
		CSD3h	−0.649 (0.023)^*^	0.669 (0.017)^*^
		CSD6h	−0.724 (0.008)^**^	0.751 (0.005)^**^
		FSD3h	−0.738 (0.006)^**^	0.799 (0.002)^**^
		FSD6h	−0.620 (0.031)^*^	0.622 (0.031)^*^
	Percentage of distance swam	CON	0.591 (0.043)^*^	−0.760 (0.004)^**^
		CSD3h	0.529 (0.077)	−0.747 (0.005)^**^
		CSD6h	0.759 (0.004)^**^	−0.683 (0.014)^*^
		FSD3h	0.710 (0.010)^**^	−0.783 (0.003)^**^
		FSD6h	0.641 (0.025)^*^	−0.657 (0.020)^*^

* Denotes significant correlation coefficients (^*^P < 0.05; ^**^P < 0.01).

#### Correlation with the mRNA levels of BDNF and Syt-1

The results revealed the hippocampal levels of BDNF mRNA in all groups were negatively correlated with the distance swam in the learning phase and positively correlated with the percentage of distance swam in the memory phase in the MWM test (*Ps* < 0.05). Furthermore, the hippocampal mRNA levels of Syt-1 in all groups were positively correlated with the distance swam and negatively correlated with the percentage of distance swam in the target quadrant (*Ps* < 0.05; Table 3). Most of the anxiety-related indicators were not correlated with the hippocampal mRNA levels of BFNF and Syt-1 in the OF and EPM tests (Table 3).

**Table 3:** Correlation between the performance in the anxiety/cognition-related tasks and hippocampal mRNA levels of BDNF and Syt-1 [*r* (*p*)].

**Tasks**	**Indexes**	**Groups**	**mRNA levels**	
			**BDNF mRNA**	**Syt-1 mRNA**
Open field test	Center time	CON	0.598 (0.014)^*^	−0.254 (0.343)
		CSD3h	0.289 (0.278)	−0.240 (0.370)
		CSD6h	0.266 (0.319)	−0.297 (0.264)
		FSD3h	0.209 (0.438)	−0.298 (0.262)
		FSD6h	−0.061 (0.821)	0.050 (0.853)
	Latency	CON	−0.170 (0.528)	0.028 (0.919)
		CSD3h	−0.239 (0.373)	−0.007 (0.979)
		CSD6h	−0.118 (0.663)	0.192 (0.475)
		FSD3h	0.161 (0.551)	−0.042 (0.876)
		FSD6h	−0.340 (0.198)	0.257 (0.336)
Elevated plus maze test	Number of entries in the open arms	CON	−0.323 (0.222)	0.227 (0.398)
		CSD3h	0.569 (0.022)^*^	−0.181 (0.501)
		CSD6h	−0.169 (0.531)	0.327 (0.216)
		FSD3h	0.261 (0.329)	0.043 (0.873)
		FSD6h	−0.058 (0.832)	0.066 (0.809)
	Time spent in the open arms	CON	0.088 (0.745)	−0.127 (0.640)
		CSD3h	−0.046 (0.866)	0.222 (0.409)
		CSD6h	0.163 (0.545)	−0.374 (0.154)
		FSD3h	−0.137 (0.614)	0.010 (0.971)
		FSD6h	−0.193 (0.473)	0.301 (0.258)
Morris water maze test	Distance swam	CON	−0.702 (0.002)^**^	0.703 (0.002)^**^
		CSD3h	−0.680 (0.004)^**^	0.602 (0.014)^*^
		CSD6h	−0.762 (0.001)^**^	0.562 (0.023)^*^
		FSD3h	−0.668 (0.005)^**^	0.566 (0.022)^*^
		FSD6h	−0.681 (0.004)^**^	0.573 (0.020)^*^
	Percentage of distance swam	CON	0.632 (0.009)^**^	−0.674 (0.004)^**^
		CSD3h	0.751 (0.001)^**^	−0.584 (0.018)^*^
		CSD6h	0.834 (0.000)^**^	−0.620 (0.010)^*^
		FSD3h	0.603 (0.013)^*^	−0.682 (0.004)^**^
		FSD6h	0.617 (0.011)^*^	−0.707 (0.002)^**^

* Denotes significant correlation coefficients (*P < 0.05; **P < 0.01).

## Discussion

Sleep disturbance is a common phenomenon in women during pregnancy (Pien and Schwab, 2004; Ross et al., 2005), and it is widely accepted that this may have deleterious effects on anxiety and cognition in offspring (Peng et al., 2016). In this study, pregnant CD-1 mice were used for studying the different patterns and durations of SD during late pregnancy for evaluating the anxious- and cognitive-related behaviors and alterations in the expression of synaptic plasticity-related proteins in offspring. Our results demonstrated that 6 h of CSD and 3/6 h of FSD in mothers can lead to increased anxiety-like behaviors and cognitive impairment in the offspring, while FSD may induce more severe cognitive impairment. Furthermore, the impaired cognitive performance in adolescent offspring may be attributed to a reduction in the expression of the *Bdnf* gene and an increase in the expression of the *Syt-1* gene in the hippocampus.

### The effects of SD during pregnancy on anxiety-like behaviors in offspring

In this study, the adolescent mice from CSD6h, FSD3h, and FSD6h groups exhibited anxiety-like behaviors in the OF and EMP tests. The findings were consistent with a previous study that showed that 2-month-old Sprague–Dawley rats, whose mothers underwent 6 h SD per day (12:00–18:00 h) during late pregnancy by gently tapping or rattling the cage, displayed anxiety-like behavior in the EPM test and novelty-suppressed feeding test (Peng et al., 2016). A previous study reporting similar findings demonstrated that the number of ultrasonic vocalizations (USVs) reduced during peak vocalization days (postnatal days 9–11) in the offspring of Wistar rats who experienced REM SD in late pregnancy. USVs in rat pups are considered to be homologous to the cries of human infants and are regarded as an expression of anxiety (Gulia et al., 2014). However, contrasting studies have demonstrated that 5 h of SD per day (9:00–14:00 h) in Wistar rats through continually enforced locomotion in late pregnancy can lead to reduced anxiety-like behaviors in pre-adolescent, adolescent, and post-adolescent offspring rats during the EPM test (Radhakrishnan et al., 2015). The discrepancy in these observations could be attributed to the different strains of rodents and variations in the duration and protocol used for SD. The present study further compared the effects of 3 and 6 h of FSD and CSD on anxiety-related behaviors, and the results demonstrated that the anxiety levels of the mice in the FSD3h, FSD6h, and CSD6h groups were similar, suggesting that continuous and fragmented SD may affect the development of the emotion-related regions of the brain to a similar extent. Interestingly, the effects of FSD induced at different durations did not have a “dose–effect relationship” on the anxiety levels. The anxiety levels of the mice in the CSD3h group were not significantly different from those in the CON group and could be attributed to the fact that the intensity of stress in the CSD3h did not reach the threshold.

### Effects of SD during pregnancy on the cognition-related behaviors of offspring

The results of this study demonstrated that the mice in the CSD6h, FSD3h, and FSD6h groups had impaired spatial learning and memory, and the FSD3h and FSD6h groups were more severe than that in the CSD6h group. Previous studies have similarly demonstrated that 6 h of SD per day (12:00–6:00 h) in Sprague–Dawley rats during early/middle/late pregnancy leads to impaired spatial learning and memory in 2-month-old offspring (Peng et al., 2016). In this study, we considered that SD in late pregnancy could be prenatal stress. The impact of prenatal stress on brain function has been associated with alterations in the regulation of the hypothalamic–pituitary–adrenal (HPA) axis of mothers, resulting in fetal exposure to high levels of corticosterone (Weinstock, 1997). Studies have demonstrated that prenatal exposure to high levels of corticosterone causes hyperactivation of the HPA axis with a concomitant reduction in the density of corticosteroid receptors in the hippocampus of adult offspring (Barbazanges et al., 1996) and mediates cognitive behavioral changes induced by prenatal stress in mice (Abdul Aziz et al., 2012). The previous study showed that prenatal stress leads to increased serum corticosterone and reduced hippocampal glucocorticoid receptors in adult offspring rats, and offspring impaired learning and memory may be related to the HPA axis (Zheng et al., 2019). Based on previous studies, we speculate that the impaired cognition of the mice in the CSD6h, FSD3h, and FSD6h groups may be attributed to the hyperactivation of the HPA axis and the noxious effects of corticosterone, which may be a potential mechanism. Some studies showed that chronic intermittent hypoxia and chronic intermittent cold stress may lead to HPA sensitization and elevated reactivity of the HPA axis (Ma et al., 2008; Girotti et al., 2011). We, therefore, speculate that the cognitive impairment observed in the FSD3h and FSD6h groups was more severe than that in the CSD6h group and might be attributed to the intermittent activation of the HPA axis induced by the repetitive awakenings of the pregnant mice. There were no differences between the CSD3h and CON groups, which could be possibly attributed to the fact that the threshold for the activation of the HPA axis was not reached. Unfortunately, we did not measure the activity of the HPA axis in this experiment, which will merit further study in our future experiments.

### Effects of SD on the hippocampal expression of *Bdnf* and *Syt-1* in offspring

Numerous studies have suggested that the hippocampal BDNF and Syt-1 are closely related to hippocampus-dependent learning and memory (Amidfar et al., 2020; Shi et al., 2020). The hippocampal levels of BDNF have been reported to be downregulated under conditions of cognitive impairment induced by prenatal restraint stress (Shang et al., 2019), perinatal ethanol exposure (Mahdinia et al., 2021), and chronic social isolation stress (Bagheri et al., 2021) in rodents. Previous studies demonstrated that prenatal restraint stress enhances the expression of Syt-1 in the hippocampus of offspring, which leads to the dendritic atrophy of pyramidal neurons by increasing the release of glutamate (Jia et al., 2010). Consistently, in this study, the hippocampal protein and mRNA of BDNF decreased and Syt-1 increased in the mice of prenatal experienced CSD6h, FSD3h, and FSD6h, and that exposure to FSD has greater effects on the hippocampal levels of both BDNF and Syt-1 than CSD. Although the effects of MSD on the hippocampal levels of BDNF and Syt-1 in the offspring were different, the mechanism underlying this difference remains to be elucidated.

### Correlation between anxiety-/cognition-related performance and expression of *Bdnf* and *Syt-1* genes

A reduction in hippocampal levels of BDNF is associated with cognitive decline, which has been reported in numerous rodent models of cognitive declines, such as Sprague–Dawley rats subjected to 48 h of SD (Wadhwa et al., 2017), Wistar rat offspring whose mothers experienced chronic mild stress during pregnancy (Guan et al., 2016), and Sprague–Dawley rats who experienced maternal separation (Ohta et al., 2017). Consistent with these results, we observed that the hippocampal levels of BDNF were correlated with the indicators in the MWM test. Syt-1 may be a stress-responsive gene that alters synaptic plasticity and is abundant in the hippocampus (Thome et al., 2001; Fox and Sanes, 2007). Our previous studies have reported that the increased hippocampal expression of Syt-1 protein and mRNA is associated with impaired learning and memory during “pathological” aging resulting from prenatal inflammatory insult (Zhang et al., 2020). Similarly, the results of the present study demonstrated that the expression of increased hippocampal *Syt-1* might be associated with impaired spatial learning and memory in adolescent mice resulting from prenatal exposure to SD.

Accumulating evidence demonstrates that apart from its role in learning and memory, the hippocampus is also the central target of the mesolimbic system that is related to the modulation of anxiety behaviors (Bannerman et al., 2004; Rezvanfard et al., 2009; Solati, 2011; Tang et al., 2019). The results of the present study demonstrated that there was no correlation between anxious behavioral indices in the OF and EPM tests and the expression of hippocampal of *Bdnf* and *Syt-1* genes in CD-1 mice whose mothers experienced SD during late pregnancy.

The present study has certain limitations, as described hereafter. First, immunohistochemistry analyses were not performed for quantitative analysis of the effect of MSD on the expression levels of BDNF and Syt-1 in the different subregions of the hippocampus. Second, the changes in the markers of the HPA axis of mothers and offspring mice were not measured, including the expression of corticosterone and glucocorticoid receptors. Third, RNA interference technologies were not used for reducing the expression of BDNF and increasing the expression of Syt-1 for verifying the targets of SD.

## Summary

In conclusion, this experiment is the first to use fragmented sleep deprivation patterns that simulate abnormal sleep patterns of pregnant women to explore its effects on offspring anxiety and cognition and the underlying molecular mechanisms, the present study demonstrated that FSD and CSD in CD-1 mice in late pregnancy can lead to increased anxiety-like behavior and cognitive impairment in adolescent offspring mice, and FSD induced worse cognitive performance than CSD. The study also demonstrated a possible link between impaired cognition and the decreased expression of the *Bdnf* gene and the increased expression of the *Syt1* gene in the hippocampus.

## Data availability statement

The original contributions presented in the study are included in the article/Supplementary material, further inquiries can be directed to the corresponding authors.

## Ethics statement

The animal study was reviewed and approved by the Association of Laboratory Animal Sciences and the Center for Laboratory Animal Sciences at Anhui Medical University (approval number: LLSC20190710).

## Author contributions

R-MW and Y-MZ designed the study, performed the behavioral tests, and prepared the manuscript. YL and Q-TW performed western blotting and real-time fluorescence-based quantitative polymerase chain reaction. Y-TW and X-YL analyzed the data and prepared the graphs. G-HC and X-WL revised the manuscript and were responsible for the completeness and accuracy of the data. All authors have read and approved the final manuscript.

## Supplementary material

Supplementary Figure 1 The learning swimming velocity and distance of the Morris water maze (MWM) test in the CSD3h and CSD6h groups. The speed and swimming distance between male and female mice were not statistically different in the CSD3h **(A,C)** and CSD6h **(B,D)** groups. Error bars = SEM. There are 8 male and 8 female mice in each treatment group.

Supplementary Figure 2 The learning swimming velocity and distance of the Morris water maze (MWM) test in the FSD3h and FSD6h groups. The speed and swimming distance between male and female mice were not statistically different in the FSD3h **(A,C)** and FSD6h **(B,D)** groups. Error bars = SEM. There are 8 male and 8 female mice in each treatment group.

Supplementary Figure 3 The vaginal smears in the diestrus phase.
