# Supplementary material for: Altered cognition and anxiety in adolescent offspring whose mothers underwent different-pattern maternal sleep deprivation, and cognition link to hippocampal expressions of Bdnf and Syt-1
Source: Front Behav Neurosci. 2022 Dec 8;16:1066725. doi: 10.3389/fnbeh.2022.1066725 (PMC9772274; doi:10.3389/fnbeh.2022.1066725)
Supplement: Supplementary Figure 1 — The learning swimming velocity and distance of the Morris water maze (MWM) test in the CSD3h and CSD6h groups. The speed and swimming distance between male and female mice were not statistically different in the CSD3h (A,C) and CSD6h (B,D) groups. Error bars = SEM. There are 8 male and 8 female mice in each treatment group. [file Data_Sheet_1.doc]

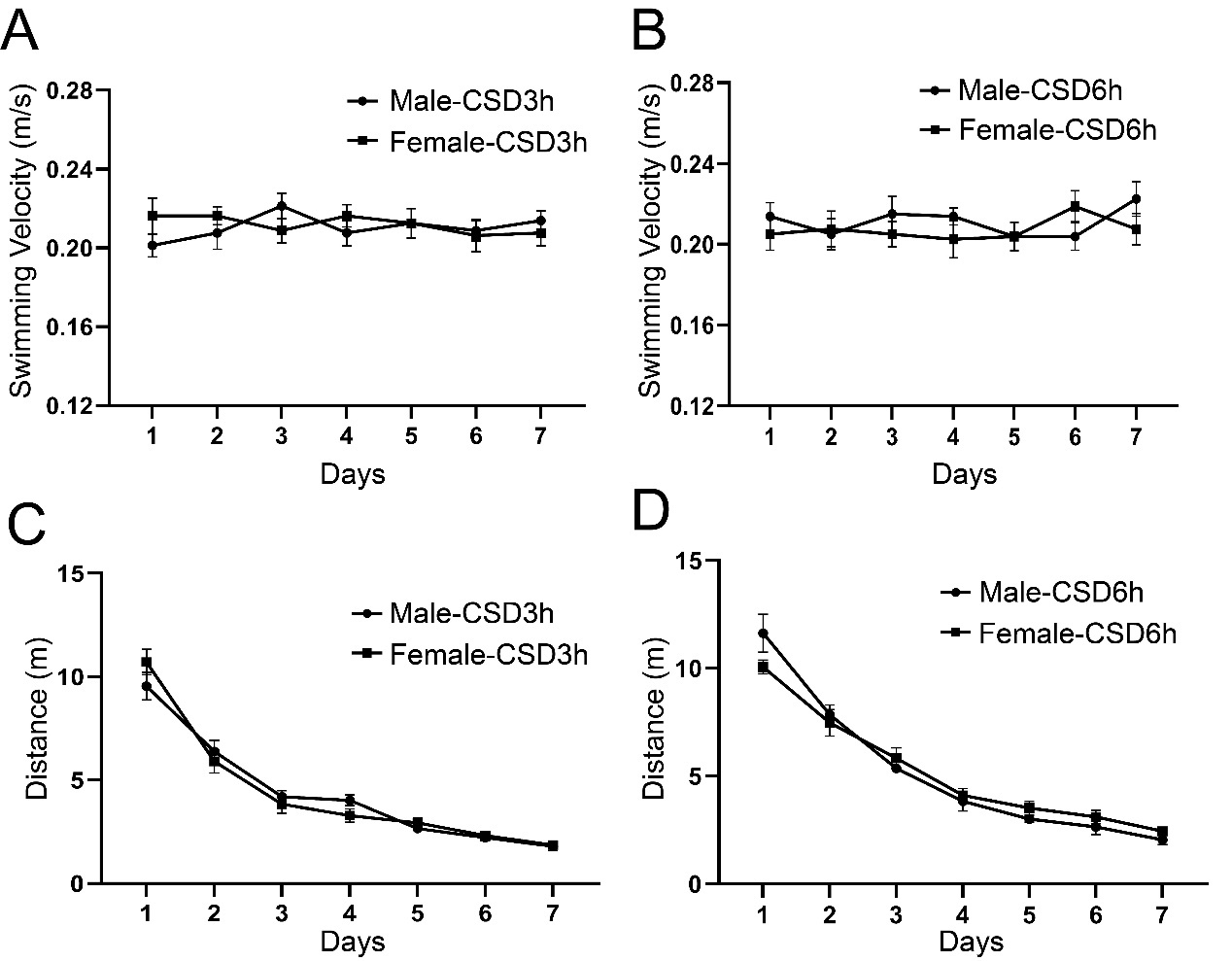


**Supplemental Figure 1.** The learning swimming velocity and distance of the Morris water maze (MWM) test in the CSD3h and CSD6h groups. The speed and swimming distance between male and female mice were not statistically different in the CSD3h (**A** and **C**) and CSD6h (**B** and **D**) groups. Error bars = SEM. There are 8 male and 8 female mice in each treatment group.


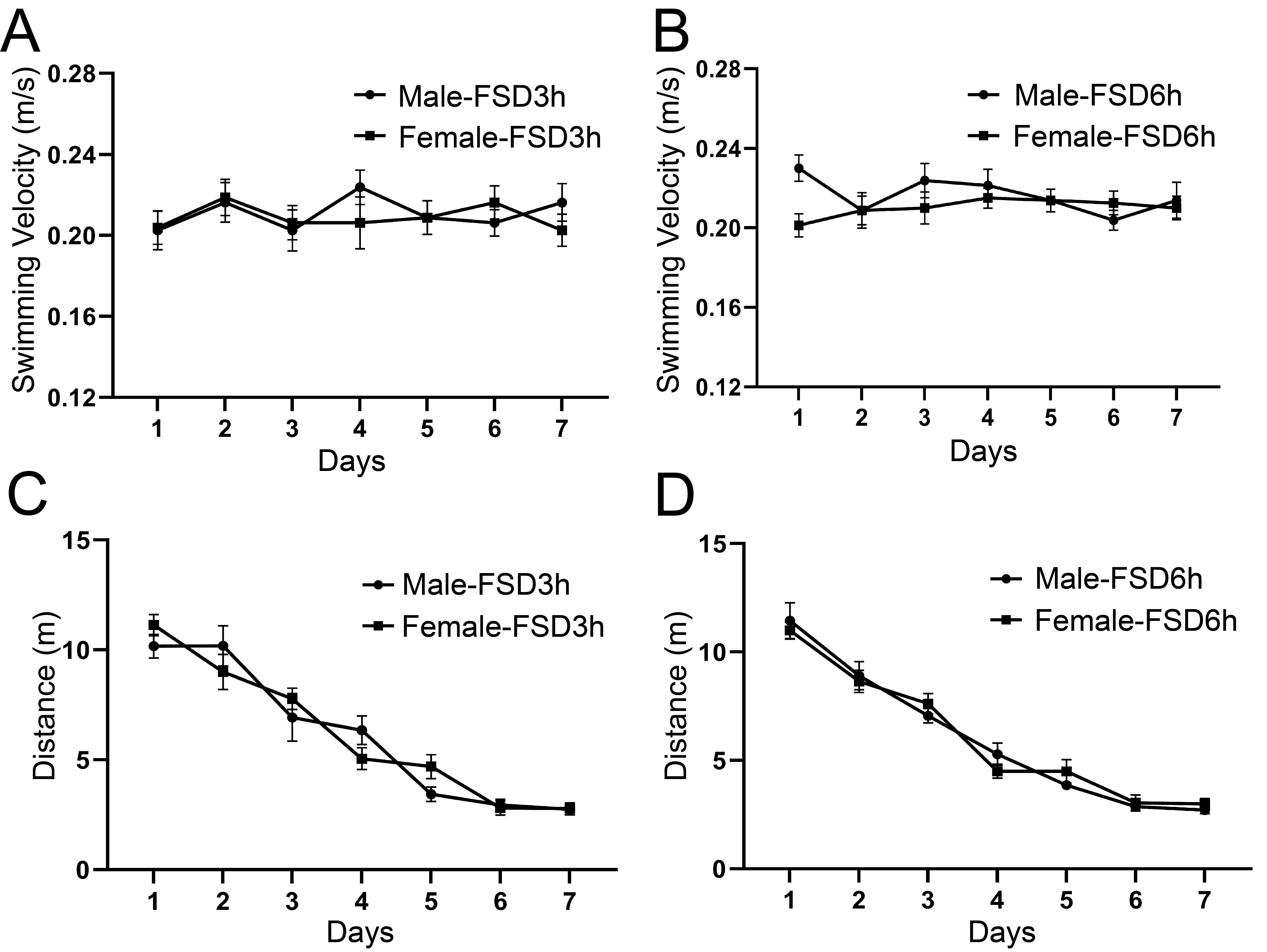


**Supplemental Figure 2**. The learning swimming velocity and distance of the Morris water maze (MWM) test in the FSD3h and FSD6h groups. The speed and swimming distance between male and female mice were not statistically different in the FSD3h (**A** and **C**) and FSD6h (**B** and **D**) groups. Error bars = SEM. There are 8 male and 8 female mice in each treatment group.
