# Supplementary figures and images for: Altered cognition and anxiety in adolescent offspring whose mothers underwent different-pattern maternal sleep deprivation, and cognition link to hippocampal expressions of Bdnf and Syt-1
Source: Front Behav Neurosci. 2022 Dec 8;16:1066725. doi: 10.3389/fnbeh.2022.1066725 (PMC9772274; doi:10.3389/fnbeh.2022.1066725)

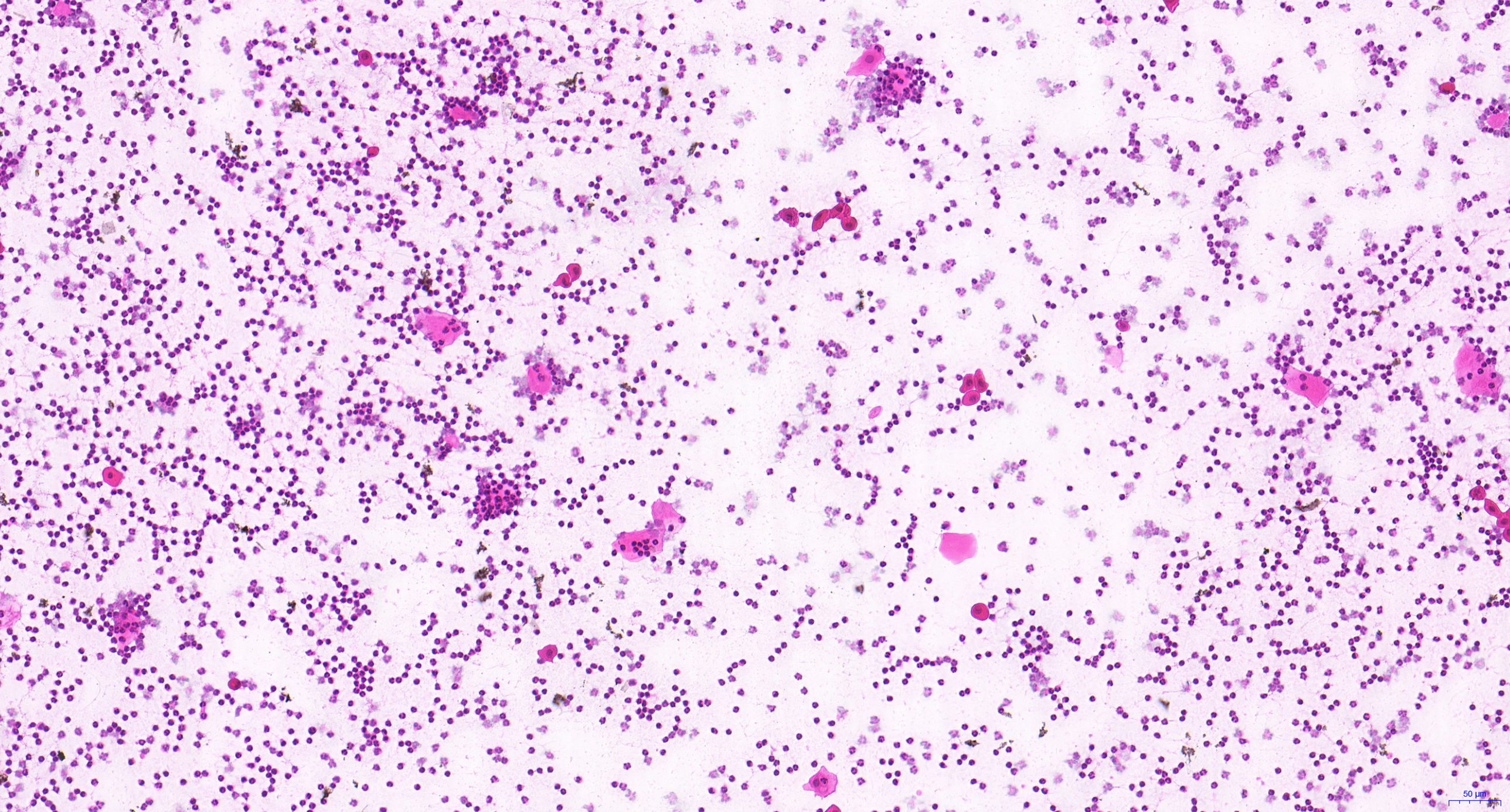

Supplement: Supplementary Figure 3 — The vaginal smears in the diestrus phase. [file Image_1.JPEG]
